# A Comparative Study on the Electrochemical Corrosion Behavior of Iron and X-65 Steel in 4.0 wt % Sodium Chloride Solution after Different Exposure Intervals

**DOI:** 10.3390/molecules19079962

**Published:** 2014-07-09

**Authors:** El-Sayed M. Sherif

**Affiliations:** College of Engineering, King Saud University, P.O. Box 800, Al-Riyadh 11421, Saudi Arabia; E-Mail: esherif@ksu.edu.sa or emsherif@gmail.com; Fax: +966-1-467-0199

**Keywords:** corrosion, chloride solutions, EIS, polarization, steel alloys

## Abstract

In this work, the results obtained from studying the anodic dissolution of pure iron and API X-65 5L pipeline steel after 40 min and 12 h exposure period in 4.0 wt % NaCl solutions at room temperature were reported. Potential-time, electrochemical impedance spectroscopy, potentiodynamic polarization, and chronoamperometric current-time at constant potential techniques were employed. It has been found that the iron electrode corrodes in the chloride test solutions faster than the API X-65 5L steel does under the same conditions. Increasing the exposure period for the electrodes from 40 min to 12 h showed a significant reduction in the corrosion parameters for both iron and steel in the 4.0 wt % NaCl solution. Results together confirmed clearly that the X-65 steel is superior to iron against corrosion in sodium chloride solutions.

## 1. Introduction

Iron and steel alloys are among the most important materials in our daily life because of their good properties and wide applications [1]. These materials have excellent weldability and good mechanical properties such as high toughness and strength. In particular, X-65 pipeline steel is considered as a high-strength low alloy (HSLA) steel that is commonly used in pipelines, off-shore rigs, agitators, pumps, tanks, *etc.* [1,2,3]. The use of X-65 steel in these many applications is partially due to its low cost compared to the higher-performing steels. A major problem usually limits the applications of pipeline steels in industry, where it suffers corrosion when exposed to aggressive media such as chloride containing and acid solutions [1,4,5,6,7,8].

The corrosion of pipeline steels represents a big issue due to the high cost and time spending in replacing, repairing and maintaining the corroded parts [4,5,6,7,8,9]. Therefore, the corrosion and corrosion inhibition of steel pipelines in harsh environments have been investigated by several researchers [4,5,6,7,8,9,10,11,12,13,14]. For example, the electrochemical corrosion behavior of API X-65 steel in the simulated oil sand slurry has been reported [10]. It was found that the corrosion records its minimum when there is no sand due to the formation of an oily layer that covers the steel surface and protects it from being attacked by any aggressive species present in the water, while the presence and the increase of the sand content increases the corrosion rate. In another work [14], the electrochemical and tension behavior of API pipeline steel was investigated in a simulated soil solution using electrochemical impedance spectroscopy and slow strain tests, where the steel was highly resistant to the stress corrosion cracking.

In previous studies [15,16,17,18,19,20,21,22], the corrosion and corrosion inhibition of different grades of steel and iron in numerous aggressive media have been reported. It has been found [15,16,17] that iron corrodes in sodium chloride solution via its dissolution into ferrous and ferric cations as follows:

Fe = Fe^2+^ + 2e^−^ (1)

Fe^2+^ = Fe^3+^ + e^−^ (2)

On other hand, the surface of iron develops oxide layers, which slows down and partially protects it from being further attacked by the chloride ions as has been confirmed by the ex and *in situ* Raman spectroscopy measurements and according to the following reactions [15,17].


Fe + ½ O_2_ + H_2_O = Fe(OH)_2_ (3)

3Fe(OH)_2_ + ½ O_2_ = Fe_3_O_4_ +3H_2_O (4)

It has also been reported [23] that iron and steels can develop up to nine different oxide phases on their surfaces. Most of these iron oxides get dissolved under the attack of chloride ions as well as the increased applied potential in the positive direction during the electrochemical polarization experiments [1,4,15,16,17].

The objective of the present work was to compare the electrochemical behavior of iron and API 5L X-65 pipeline steel in 4.0 wt % NaCl solutions at room temperature. The aim was extended to investigate the effect of increasing the immersion time from 40 min to 12 h on the anodic dissolution as well as the pitting corrosion of iron and X-65 steel in the aerated stagnant chloride solutions. The study was carried out using the traditional electrochemical measurements such as open-circuit potential, potentiodynamic polarization, and chronoamperometric current-time at constant potential, −0.350 V *vs.* Ag/AgCl. The EIS Nyquist plots were also employed to determine the kinetic parameters for electron transfer reactions at the electrode/electrolyte interface.

## 2. Result and Discussion

### 2.1. Potentiodynamic Polarization Measurements

**Figure 1:**
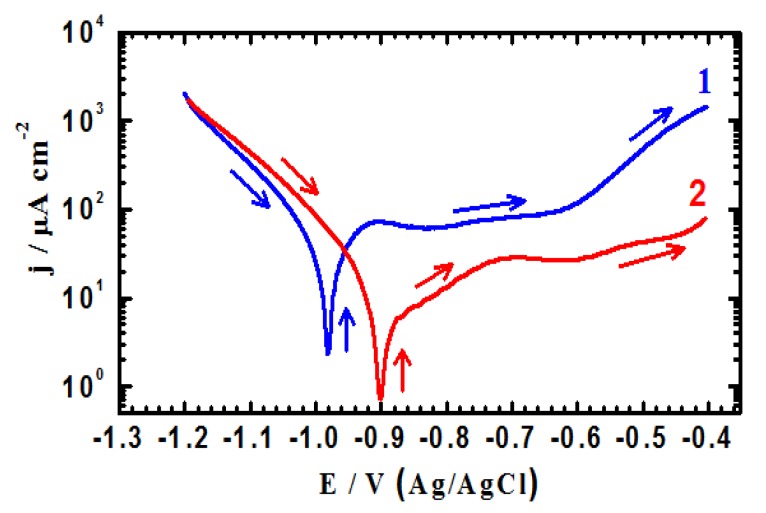
Potentiodynamic polarization curves for (1) pure iron and (2) API X-65 steel after their immersions for 40 min in 4.0 wt % NaCl solutions.

**Figure 2:**
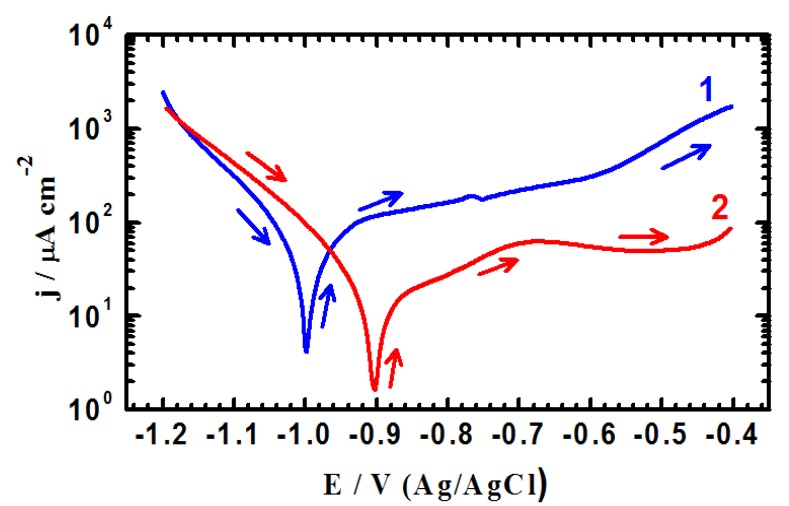
Potentiodynamic polarization curves for (1) pure iron and (2) API X-65 steel after their immersion for 12 h in 4.0 wt % NaCl solutions.

Potentiodynamic polarization testing were conducted to measure the corrosion parameters such as corrosion potential (E_Corr_), corrosion current (j_Corr_), polarization resistance (R_P_) and corrosion rate (R_Corr_), for iron and API X-65 steel after their immersion in the chloride test solution for 40 min and 12 h. The potentiodynamic polarization curves obtained for (1) iron and (2) X-65 steel after their immersion in 4.0 wt % NaCl solutions for 40 min are shown in Figure 1. The same measurements were carried out for iron and steel after 12 h immersion in the test solution and curves are shown in Figure 2. The values of cathodic (β_c_) and anodic (β_a_) Tafel slope, corrosion potential (E_Corr_), corrosion current density (j_Corr_), polarization resistance (Rp), and corrosion rate (R_Corr_) obtained for iron and X-65 steel electrodes from the polarization curves shown in Figure 1 and Figure 2 are listed in Table 1. The values of (β_c_) and (β_a_) were determined after at least 50 mV away from E_Corr_ and at least one decade of current densities (j_Corr_). The values of E_Corr_ and j_Corr_ were obtained from the extrapolation of anodic and cathodic Tafel lines located next to the linearized current regions. The values of polarization resistance, Rp, for iron and X-65 steel were calculated as reported in our previous work [15,16,24,25] corrosion as follows:

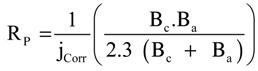
(5)

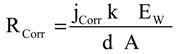
(6)
where, k is a constant that defines the units for the corrosion rate (=3272 mm/(amp.cm.year)), E_W_ the equivalent weight in grams/equivalent of iron alloy (E_W_ = 27.93 grams/equivalent), d the density in gcm^−3^ (=7.86), and A the area of the exposed surface of the electrode in cm^2^.

It has been reported [15,16,17,18,19] that the anodic reaction of iron and steel alloys occurs via the corrosion of its surface on two steps as can be seen from Equations (1) and (2). At the same time, the cathodic reaction for these materials in aerated near neutral chloride solutions is the oxygen reduction that consumes the released electrons from the anodic reaction according to the following equation [15,16,17].


O_2_ + 2H_2_O + 4e^−^ = 4OH^−^ (7)

**Table 1:** Parameters obtained from potentiodynamic polarization curves shown in Figure 2 and Figure 3 for iron and API X-65 steel after 40 min and 12 h immersion in 4.0 wt % NaCl solutions.

Material/Time	Parameter					
	β_c_/mVdec^−^^1^	j_Corr_/μAcm^−2^	E_Corr_/mV	β_a_/mVdec^−^^1^	R_p_/Ωcm^2^	R_Corr_/mmy^−1^
Iron (40 min)	70	28	−985	80	580	0.326
X-65 (40 min)	90	15	−823	125	1517	0.174
Iron (720 min)	95	18	−907	115	1257	0.209
X-65 (720 min)	105	10	−862	140	2609	0.116

It is clearly seen from Figure 1 that the anodic and passivation currents for iron were higher than those recorded for X-65 steel. Moreover, the values of j_Corr_ and R_Corr_ were higher, the value of Rp was smaller, and the value of E_Corr_ was more negative than those obtained for the X-65 as indicated by the parameters listed in Table 1. The values of E_Corr_ were certainly affected by the strong cathodic polarization for iron and reflected in turn on a more active iron surface with more anodic currents compared to X-65 steel. The higher anodic currents for iron indicated that the iron suffers more corrosion via its dissolution into ferrous and ferric cations as represented by Equations (1) and (2). The wider passivation region with lower passivation current and less negative corrosion potential proved also that the X-65 had higher corrosion resistance compared to iron in the chloride solution. It is worth to mention that the passive region appeared on the anodic branch of the polarization curves for iron and steel was due to the formation of iron oxides and/or corrosion products on their surfaces as shown by Equations (3) and (4). Where, the formed ferrous hydroxide reacted with more oxygen to form the top layer of magnetite corrosion product, Fe_3_O_4_. The presence of such oxide partially protects the iron surface from further dissolutions and led to the appearance of the passive region. Moreover, the higher Rp value for X-65 steel than iron revealed that the surface of X-65 steel was more passivated against corrosion than the iron surface. The lower values of j_Corr_ and R_Corr_ recorded for X-65 steel further confirmed that the steel did not suffer severe corrosion compared to the more corroded iron surface. The polarization data after 40 min immersion in 4.0 wt % NaCl solutions thus concluded that the dissolution of iron was greater than that of API 5 L X-65 pipeline steel at the same conditions.

Increasing the immersion time for iron and API X-65 steel in 4.0 wt % NaCl solutions to 12 h before measurements, Figure 2, significantly shifted the values of E_Corr_ for both iron and steel to the less negative values and highly reduced the values of j_Corr_ and R_Corr_, in addition to the remarkable increased values for the polarization resistance, Rp. Increasing the immersion time of iron and steel thus decreased their corrosion via developing and thickening a layer of corrosion products. The formation of these layers provides a partial protection for the surface from being attacked by the aggressive chloride ions and leads in turn to decreasing the corrosion parameters. The polarization data thus indicate that iron corrodes in 4.0 wt % NaCl solutions faster than API X-65 does and increasing the immersion time decreases the chloride ions attack on their surfaces.

### 2.2. Chronoamperometric Current-Time (CT) Measurements

**Figure 3:**
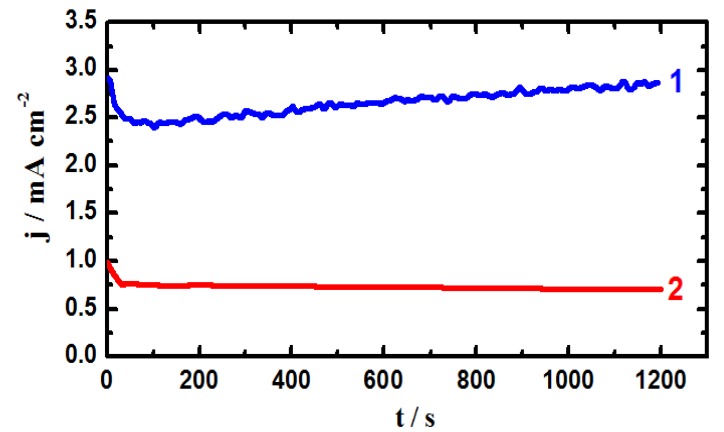
Chronoamperometric current-time curves obtained at −0.350 V *vs.* Ag/AgCl for (1) iron and (2) API X-65 steel electrodes after 40 min immersions in 4.0 wt % NaCl solutions.

**Figure 4:**
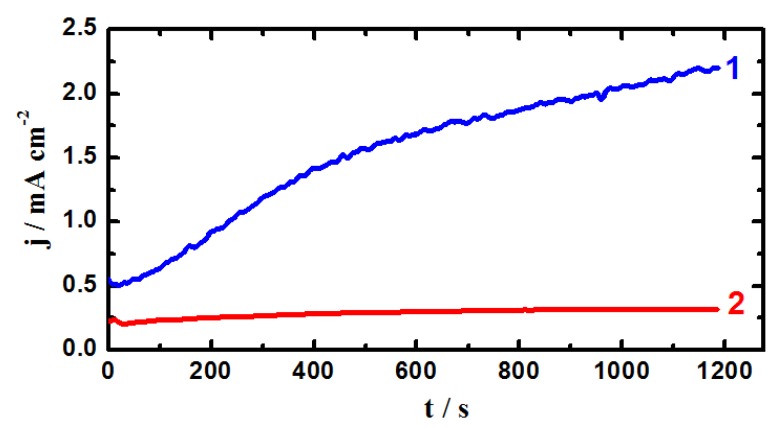
Chronoamperometric current-time curves obtained at −0.350 V *vs.* Ag/AgCl for (1) iron and (2) API X-65 steel electrodes after 12 h immersions in 4.0 wt % NaCl solutions.

In order to shed more light on the effect of applying an active anodic potential on both uniform and pitting corrosion of iron and X-65 steel and also increasing the time of immersion from 40 min to 12 h in the chloride solutions, the CT experiments were carried out. Figure 3 shows the CT curves obtained at −0.35 V *vs.* Ag/AgCl for (1) iron and (2) X-65 steel electrodes, respectively after 40 min immersions in 4.0 wt % NaCl solutions. The CT curves were also recorded for (1) iron and (2) X-65 steel electrodes after 12 h of their immersion in the test solution as shown in Figure 4. The recorded current for iron after 40 min immersion, Figure 3 (curve 1) showed a gradual increase in its value with increasing time of the experiment. On the other hand, applying this potential value, −0.35 V, on the API X-65 pipeline steel produced low current that decreased with increasing time.

The increase of current for iron with time indicates that iron suffers severe pitting corrosion at −0.35 V just after its immersion in 4.0 wt % NaCl solutions for 40 min. It has been reported [1,4,9,10,11,15,16,17] that the chloride ions present in the solution aggressively attack the iron surface at anodic active potential leading to a continuous dissolution of iron through severe uniform and pitting corrosion according to the following reactions [1,4,15,16,17].


Fe + Cl^−^ = Fe(Cl^−^)_surface_ (8)

Fe(OH)_surface_ + Fe(Cl^−^)_surface_ = Fe + FeOH^+^ + Cl^−^ + 2e^−^ (9)

FeOH^+^ + H^+^ = Fe^2+^_solution_ + H_2_O (10)

Fe _Surface_ + 2Cl _Solution_ = FeCl_2 Surface_ + 2e^−^ (11)

FeCl_2 Surface_ = FeCl_2interface_ = FeCl_2 Solution_ (12)

The formed ferrous chloride on the iron surface does not last long and coverts into the interface before leaving the iron surface and dissolving in the solution. The chloride ions presented in the solution further target the formed ferrous chloride on the iron surface as well as in the solution to form ferric chloride as follows [26,27,28,29,30]:

FeCl_2 Surface_ + Cl^−^_(aq)_ = FeCl_3 Surface_ + e^−^ (13)

FeCl_3 Surface_ = FeC1_3 (interface)_ → FeCl_3 Solution_ (14)

The formation of these iron chloride compounds further accelerates the dissolution of iron itself by attacking its surface either after the chloride compounds leached out from the surface to the solution or beneath the corrosion products leading to the formation of pits on its surface as can be explained by Equations (5)–(7) [26,27,28,29,30]. The presence of some inclusions in pure iron might also have helped in the occurrence of its pitting corrosion and the increase of its severity with time.

The CT behavior of API X-65 steel tells that these aforementioned reactions do not seem to occur on its surface compared to those obtained for iron at the same applied potential and immersion time. The CT experiments for X-65 steel thus reveal that the steel did not suffer neither uniform nor pitting corrosion and confirm that the pitting potential of X-65 steel grade is more positive than −0.35 V *vs.* Ag/AgCl and its surface was more passivated compared to iron in 4.0 wt % NaCl solution.

The CT curves obtained for iron and steel after 12 h immersion in the chloride test solution, Figure 4, showed almost the same behavior like those ones after 40 min immersion, Figure 3. The only difference was the lower values obtained for the absolute currents for both iron and steel. This aggress with the data obtained by polarization measurements, Figure 1 and Figure 2, and confirms that elongating the immersion time for these materials before measurements allows their surfaces to develop a top layer of oxides and/or corrosion products that get thicker with time and thus resist the chloride ions attack, which in turn lowers the obtained current values.

### 2.3. Open-Circuit Potential (OCP) vs. Time Measurements

**Figure 5:**
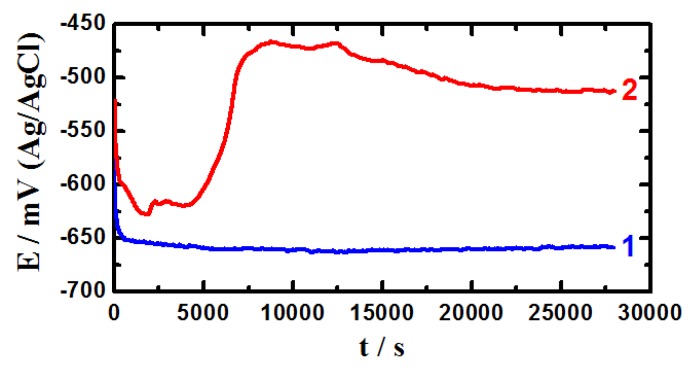
Potential-time curves obtained for (1) pure iron and (2) API X-65 5 L pipeline steel, respectively in 4.0 wt % NaCl solutions.

Figure 5 shows the potential-time curves obtained for (1) pure iron and (2) API 5 L X-65 pipeline steel, respectively in 4.0 wt % NaCl solutions. It is seen from Figure 5 that the initial potential of iron (curve 1) increased towards the more negative values due to the dissolution of a preformed air oxide film. The potential then slightly shifted in the more negative direction with increasing time till the end of the experiment. This more negative shift might have resulted from the dissolution of iron by the chloride ions attack. While, the slight positive shift in the potential of iron with time was due to the formation of corrosion products and/or an oxide film on the iron surface; these provided partial protection for iron and does not completely prevent its dissolution.

On the other hand, the API X-65 steel (curve 2) showed similar potential behavior in the first 1,800 s, where the potential was decreasing in the more negative direction with time. This steel potential-time behavior indicates that the chloride ions presented in the solution attacked the performed air oxide film causing its dissolution. Increasing the immersion time, ≥1,800 s, positively shifted the free potential for X-65 steel, which was most probably due to the formation of an oxide film. Further increasing in time, >4000 s, led to an abrupt shift in the noble potential direction, which resulted by the thickening of the formed oxide and/or the development of corrosion products on the steel surface. Such oxide and corrosion products provide partial protection against corrosion of steel in 4.0 wt % NaCl solution. The potential-time behavior for iron and steel indicates that the surface of the API X-65 steel is more corrosion resistant than that obtained for iron at the same conditions. This agrees with the data obtained by polarization and chronoamperometric current-time that X-65 steel is more passivated compared to pure iron in 4.0 wt % NaCl solutions and this behavior increases with increasing the immersion time.

### 2.4. Electrochemical Impedance Spectroscopy (EIS) Measurements

**Figure 6:**
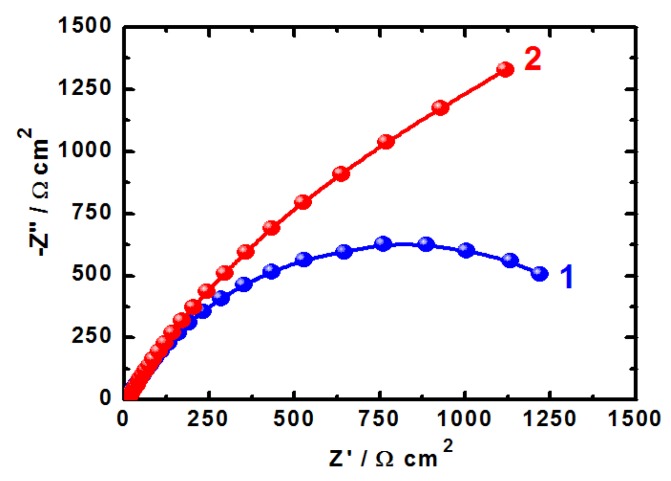
EIS Nyquist for (1) pure iron and (2) API X-65 steel at corrosion potential after 40 min immersion in 4.0 wt % NaCl solutions.

**Figure 7:**
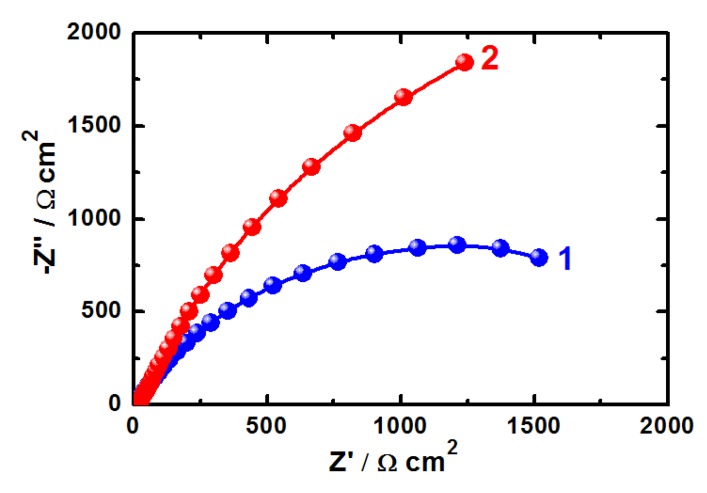
EIS Nyquist plots for (1) pure iron and (2) API X-65 steel at corrosion potential after 12 h immersion in 4.0 wt % NaCl solutions.

**Figure 8:**
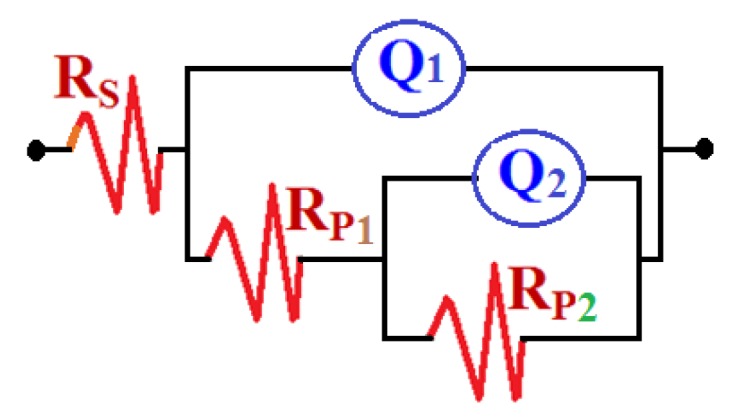
The equivalent circuit model used to fit the EIS experimental data.

**Figure 9:**
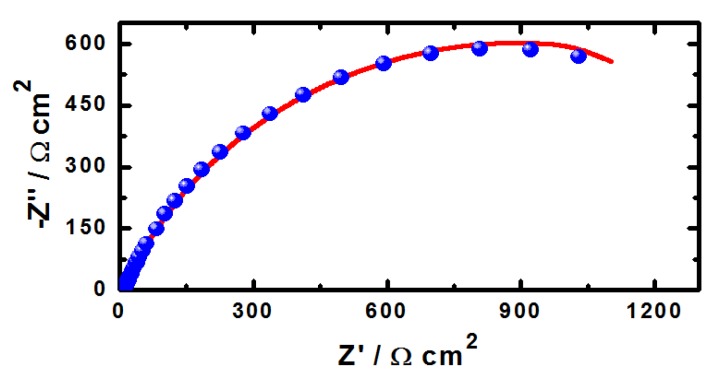
EIS Nyquist plot for pure iron after 40 min immersion in 4.0 wt % NaCl solution; symbols represent the measured data and solid line represents the best fit using the equivalent circuit shown in Figure 8.

EIS measurements have been used in studying corrosion and corrosion inhibition of metals and alloys in different corrosive media [15,16,17,18,19,20,31]. The Nyquist plots obtained for (1) iron and (2) API X-65 steel electrodes at their open-circuit potential after their immersion in 4.0 wt % NaCl solutions for 40 min and 12 h are shown in Figure 6 and Figure 7, respectively. The spectra represented in these figures were analyzed by best fitting to the equivalent circuit model shown in Figure 8. In order to confirm that the used circuit represents the best fit, the EIS Nyquist was plotted for iron after 40 min immersion in the chloride solution and shown in Figure 9; symbols represent the measured data and solid line represents the best fit using the equivalent circuit shown in Figure 8. The EIS parameters obtained by fitting this circuit are listed in Table 2.

**Table 2:** EIS parameters obtained by fitting the Nyquist plots shown in Figure 6 and Figure 7 with the equivalent circuit shown in Figure 8 for iron and API X-65 steel after 1 and 12 h immersion in 4.0 wt % NaCl solutions.

Material/Time	Parameter						
	R_S_/Ω cm^2^	Q1 (CPEs)		R_P1_/Ω cm^2^	Q2 (CPEs)		R_P2_/Ω cm^2^
		Y_Q1_/μF cm^−2^	*n*		Y_Q2_/μF cm^−2^	*n*	
Iron (40 min)	6.47	7.44	0.93	795	6.17	0.73	1167
X-65 (40 min)	7.73	5.51	1.0	897	4.98	0.74	1293
Iron (720 min)	6.75	6.35	1.0	1102	4.69	0.72	1524
X-65 (720 min)	8.87	4.04	1.0	1223	3.74	0.80	2415

The parameters of the equivalent circuit model shown in Figure 8 can be defined according to the usual convention as follows; R_S_ represents the solution resistance, Q1 and Q2 are the constant phase elements (CPEs), and Rp1 and Rp2 are the polarization resistances. It is seen from Figure 6 and Figure 7 that both iron and steel showed only one semicircle and the diameter is wider for steel than it is for iron. This indicates that the resistance against corrosion for API X-65 steel is higher than for iron. The values of R_S_, Rp1 and Rp2 (see Table 2) for steel recorded also higher values compared to those listed for iron due to the increase of the corrosion resistance for the steel surface than the iron one. The constant phase elements (Q, CPEs) with its n values close to 1.0 for all samples represent double layer capacitors with little porosities [15,16,17,18,19,20]. This is because depending on the value of *n*, CPE can represent resistance (Z(CPE) = R, *n* = 0), capacitance (Z(CPE) = Cdl, *n* = 1) or Warburg impedance for (*n* = 0.5). Therefore, the CPE for iron and steel is substituted for the capacitor to fit the semicircle more accurately. The admittance and the impedance of a CPE can be defined according to the following equations, respectively [32].

Y_CPE_ = Y_0_ (jω)^n^ (15)

Z_CPE_ = (1/Y_0_) (jω)^−n^ (16)

where, Y_0_ is the modulus, ω is the angular frequency, and n is the phase.

Increasing the immersion time to 12 h before measurements, Figure 7, increased the size of the diameter of the semicircle both for iron, curve 1 and API 5 L X-65 pipeline steel, curve 2 compared to their sizes after 40 min immersion in the chloride solution, Figure 6. It is also seen from Figure 7 that the diameter of the semicircle plotted for X-65 was much wider than that drawn for iron. The parameters listed in Table 2 showed that the values of the resistances, R_S_, Rp1 and Rp2 obtained after 12 h immersion in 4.0 wt % NaCl solution recorded higher values compared to the ones obtained after only 40 min and their values with X-65 were higher than those for iron at the same conditions. Moreover, the CPEs recorded lower value with X-65 than that obtained with iron and further decreases were provided with increasing the immersion time for 40 min to 12 h. The EIS measurements thus confirm that X-65 steel showed better corrosion resistance and higher surface passivation than iron after the same immersion time in 4.0 wt % NaCl solutions and that elongating the exposure time period before measurement to 12 h decreases the corrosion of both materials. The EIS data are in good agreement with the results obtained by potentiodynamic polarization, chronoamperometry, and OCP measurements. All data confirmed that iron is more susceptible to uniform and pitting corrosion compared to the API 5 L X-65 pipeline steel in 4.0 wt % NaCl solutions and the increase of immersion time decreases the severity of the chloride ions against corrosion due to the oxide film and corrosion product thickening on the surface of iron and steel.

## 3. Experimental Section

### 3.1. Chemicals and Materials

A stock solution of 4.0 wt % sodium chloride (NaCl, 99%, Merck, Johannesburg, South Africa) was prepared by dissolving 40 g of NaCl in 1 L glass flask. An iron rod (Fe, 99.99%, Goodfellow, Huntingdon, UK, 1.0 cm in diameter) was purchased from Aldrich, Dorset, UK. An iron rod (Fe) of 99.98% purity and having a diameter of 9.5 mm was purchased from Goodfellow. An API X-65 5 L steel electrode had a square shape and a total surface area of 1 cm^2^. The chemical composition of the API steel was as follows, 1.5 wt % Mn, 0.35 wt % Si, 0.15 wt % P, 0.10 wt % C, 0.015 wt % N, 0.005 wt % S, and the balance was Fe.

### 3.2. Electrochemical Cell

An electrochemical cell with a three-electrode configuration was used; the iron and API X-65 steel rods; a platinum foil, and an Ag/AgCl electrode (in 3.0 M KCl solution) were used as the working, counter, and reference electrodes, respectively. Before immersed in the chloride solution, the surface of the working electrode was ground successively with metallographic emery paper of increasing fineness up to 1,200 grit. The surface was further cleaned using doubly-distilled water, degreased with acetone, washed using doubly-distilled water again and finally dried with dry air.

### 3.3. Electrochemical Corrosion Measurements

All the electrochemical experiments were carried out using an Autolab electrochemistry setup (Metrohm Autolab B.V., Amsterdam, the Netherlands) operated by the general purpose electrochemical software (GPES) version 4.9. The potentiodynamic polarization curves were obtained by scanning the potential in the forward direction from −1.2 V to −0.40 V *vs.* Ag/AgCl at a scan rate of 1.0 mV/s. Chronoamperometric current-time measurements were collected by fixing the potential of the working electrode at −0.350 V *vs.* Ag/AgCl. The open circuit potential (OCP) experiments were carried out for about 8.0 h. The electrochemical impedance spectroscopy measurements were performed at OCP over a frequency range of 100 kHz to 100 mHz, with an AC wave of ± 5 mV peak-to-peak overlaid on a DC bias potential. The impedance data were collected using the Powersine software at a rate of 10 points per decade change in frequency.

## 4. Conclusions

A comparative study on the electrochemical corrosion behavior of iron and API 5 L X-65 pipeline steel grade in aerated stagnant solutions of 4.0 wt % NaCl was reported. The work was carried out using a variety of electrochemical measurements, including potentiodynamic polarization, chronoamperometric current-time (CT) at constant potential, open-circuit potential (OCP) and electrochemical impedance spectroscopy (EIS). It has been found that iron records higher anodic, cathodic and corrosion currents and corrosion rate with lower corrosion resistance compared to the API X-65 steel at the same conditions. CT experiments at −0.35 V *vs.* Ag/AgCl indicated that iron suffers both pitting and uniform corrosion, while X-65 steel does not because the latter recorded very low absolute currents with time. The OCP test showed that the potential of iron shifts toward the more negative values, unlike the potential of X-65 that shifts to the less negative direction and the potential difference between them was circa 0.15 V after about 60 min in the chloride solution. EIS data agreed with the polarization, CT, and OCP results showing that the API 5 L X-65 pipeline steel showed more surface and polarization resistances and its surface was more passivated compared to the surface of iron. All results were consistent with each other confirming clearly that iron suffers both uniform and pitting corrosion and that the X-65 steel has high corrosion resistance. The increase of the immersion time from 40 min to 12 h increased the corrosion resistance for both iron and steel in 4.0 wt % NaCl solution.
